# The Chemistry, Pharmacology and Therapeutic Potential of the Edible Mushroom *Dictyophora indusiata* (*Vent* ex. *Pers.*) Fischer (Synn. *Phallus indusiatus*)

**DOI:** 10.3390/biomedicines7040098

**Published:** 2019-12-12

**Authors:** Solomon Habtemariam

**Affiliations:** Pharmacognosy Research Laboratories & Herbal Analysis Services UK, University of Greenwich, Chatham-Maritime, Kent ME4 4TB, UK; s.habtemariam@herbalanalysis.co.uk; Tel.: +44-208-331-8302

**Keywords:** *Dictyophora indusiata*, *Phallus indusiata*, *polysaccharide*, beta-glucans, mushrooms, immunotherapy, cancer, neurodegenerative diseases, obesity, hyperlipidemia

## Abstract

*Dictyophora indusiata* (Vent. Ex. Pers.) Fischer or *Phallus indusiatus* is an edible member of the higher mushroom phylum of Basidiomycetes. Known for its morphological elegance that gave it the names bridal veil fungus, veiled lady or queen of the mushrooms, it has numerous medicinal values that are beginning to be acknowledged through pharmacological efficacy studies. In an attempt to promote research on this valuable natural resource, the present communication aims to provide a comprehensive review of the chemistry, pharmacology and potential therapeutic applications of extracts and compounds isolated from *D. indusiata*. Of the bioactive compounds, the chemistry of the polysaccharides as major bioactive components primarily the *β*-(1→3)-D-glucan with side branches of *β*-(1→6)-glucosyl units are discussed, while small molecular weight compounds include terpenoids and alkaloids. Biochemical and cellular mechanisms of action from general antioxidant and anti-inflammatory to more specific signaling mechanisms are outlined along with potential applications in cancer and immunotherapy, neurodegenerative and chronic inflammatory diseases, etc. Further research areas and limitations of the current scientific data are also highlighted.

## 1. Introduction

**Figure 1:**
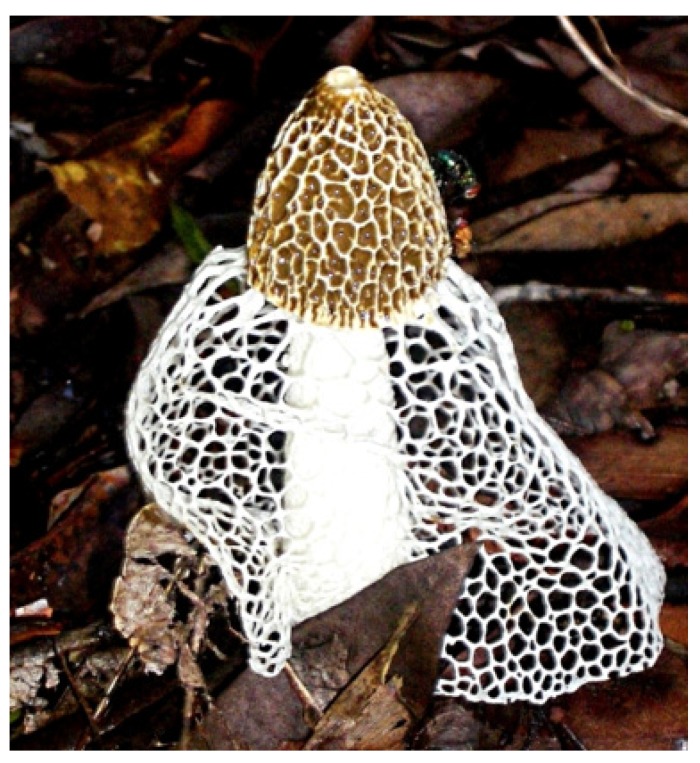
The fruiting body of *Dictyophora indusiata.* Image curtesy of Wikipedia (https://commons.wikimedia.org/wiki/File:Dictyophora_indusiata._Cooktown,_Australia._2010.JPG).

*Dictyophora indusiata (Vent.)* Fisch. is an edible and medicinal mushroom that belongs to the family *Phallaceae* of the Agaricomycetes class (phylum Basidiomycetes) of fungi. In recent taxonomic literature, the synonym *Phallus indusiata Vent*. is the accepted name for the fungus though nearly all the scientific literature so far is available under the name entry of *D. indusiata*. As a saprophytic fungus, it grows in well-rotted woody trunk or rich soil of tropical Africa, Asia, Australia and the Americas. Its food and medicinal value are however much appreciated in the far eastern countries such as China where it grows on the wet roots of bamboo groves and in forests. Its common local names mainly in China and Japan include bamboo mushrooms, bamboo pith, long net stinkhorn, crinoline and stinkhorn basket, but perhaps the names most vividly associated with the morphologically distinctive feature of the fungus are bridal veil fungus, veiled lady or queen of the mushrooms. As shown in Figure 1, the fruiting body of *D. indusiata has three macroscopic features:* conical cap, a stalk and an indusium net-like white veil that hangs from the head/cap down to cover the leg/stalk. Hence, the name-veiled lady appears to be given to describe the skirt like appearance of the elegant fruiting body (Figure 1).

Like other edible mushrooms, *D. indusiata* sourced both from the wild and commercial sources has nutritional value and its protein, carbohydrates, and dietary fibre contents have been extensively studied [1,2]. Likewise, the amino acids, vitamins and inorganic elements composition of *D. indusiata* have been reported [3]. The composition of carbohydrates can reach up to 47% of the dry weight of which the soluble polysaccharides are about 38%, while crude fibres and crude proteins contents are about 29% and 6% respectively [1]. In this communication, the emphasis is on the compositions of the fruiting body far beyond nutritional source with a focus on their pharmacologically active components and their therapeutic implications. In this connection, considerable attention has been given to the major components of the fungus, the polysaccharides, while small molecular weight drug-like molecules such as terpenoids and alkaloids have not been extensively studied. The chemistry and biological significance of all the bioactive compounds known from D. indusiata are herein scrutinised.

## 2. Overview of Chemistry

### 2.1. Polysaccharides

**Figure 2:**
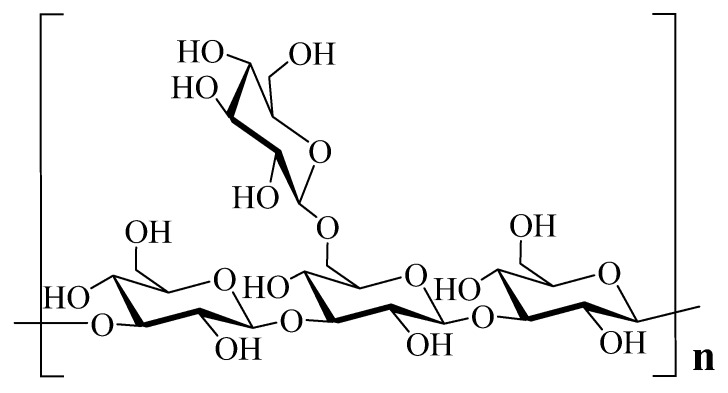
Backbone structure of polysaccharides as 1, 3-*β*-glucans.

Wang et al. [4] established the structure of a polysaccharide from the fruiting bodies of *D. indusiata* as a homogeneous *β*-(1→3)-D-glucan with side branches of *β*-(1→6)-glucosyl units. The molecular mass and total sugar content were 536 kDa and 97.6% respectively. Various molecular weight polysaccharides have been identified however and a range of 801–4656 kDa for six polysaccharides have been described [1]. The general backbone of the polysaccharides structure of *D. indusiata* is now well established as a (1→6)-branched, (1→3)-*β*-d-glucan and is as depicted in Figure 2. Earlier studies as far back to the year 1982 established this structure from the water-soluble alkali extract [5]. Different components of the acid soluble and alkali soluble polysaccharides fractions with variable composition of the sugar monomeric units such as Glucose (Glc) and fructose (Fru) have been established [6]. In one analysis, the crude polysaccharide extract was shown to have a monosaccharide molar composition of Glc, mannose (Man), and galactose (Gal) as 59.84%, 23.55%, and 12.95%, respectively [7]. While Man was detected as Man (2→1) branch in some, the molar ration of the Glc (1→6) linkage have also been identified as a source of variability among the polysaccharides [1]. Further variations of the water or sodium carbonate polysaccharide extracts include a (1→3)-*α*-D-mannan, branched (1→3)-*β*-D-glucan and the location of the *O*-acetylation of the (1→3)-*α*-D-mannan [8,9,10,11,12]. Phosphorylated polysaccharides could also be obtained from the mushroom as shown by Deng et al. [13] in their work on the physicochemical characterization of polysaccharides obtained from a water-insoluble crude polysaccharides.

Another interesting structural feature of the polysaccharides is the triple helical chain conformation of some in water. For example, Wang et al. [14] isolated these polysaccharides as water-soluble glucans while Deng et al. [15] further shown the biological activity of a triple helical chain polysaccharide (see also Table 1). Methods for quantitative analysis of 1, 3-*β*-glucans have also been developed of which the fluorometric assay based on aniline blue dye described by Fu et al. [16] was a good example. Using this method, they have shown the maximum extraction yield of the water soluble 1, 3-*β*-glucans as 1.20% and total polysaccharides as 5.41%. Wu et al. [17] further developed an enzyme-assisted extraction methodology for the water-soluble polysaccharides from *D. indusiata*. For the three kinds of enzymes (cellulase, papain and pectolyase) employed, they established an optimum extraction temperature of 52.5 °C, extraction time 105 min and pH of 5.25. Considering the predicted polysaccharide yield of 9.87%, the enzyme-assisted extraction yield of 9.77 ± 0.18% appear to be an exhaustive extraction method. On the other hand, Liu et al. [18], employed response surface methodology to maximise the yield of polysaccharides extraction from the fruiting body of *D.* *indusiata*. Under the optimum extraction time of 2.1 h, solid to liquid ratio of 1:37, and extraction temperature of 92 °C, they were able to enhance the yield to 15.95%. Water-soluble polysaccharides have also been routinely extracted by ultrasonic extraction (e.g., [16]).

*D.indusiata* polysaccharides are normally prepared by water extraction followed by centrifugation, removal of proteins and alcohol precipitation. As a general approach, the dried fruiting body may be subjected to organic solvent extraction (e.g., reflux with methanol) to remove lipophilic components and the remaining part can be extracted by either of the methodologies mentioned above. In many cases, hot or boiling water extraction by distilled/deionized water for up to 2 h are performed for initial extraction. After filtration and removal of water (rotary evaporator or freeze drying), proteins are removed (e.g., Sevag method, [19]). The crude polysaccharides are usually obtained by precipitation with ethanol. In many experiments cited in this communication (see Table 1 and Table 2), such crude polysaccharides with some degree of compositional analysis are assessed for biological activity. On the other hand, several methods have been employed for the isolation of purified polysaccharides from the fruiting body. The isolation of the polysaccharide named Dectin-1, for example, was based on gel filtration chromatography. The resulting polysaccharide with the backbone of (1→3)-*β*-glucan (1→6)-*β*-glucan side chain residues had the molecular weight of 650 kDa, and the total sugar and uronic acid contents were 95.67% and 0.63% respectively [20]. A previous study by the same authors also employed Sephadex G-200 for the purification of a *β*-glucan polysaccharide from *D. indusiata* [21]. Anion-exchange chromatography was also employed by Zhang et al. [19] to isolate the acidic polysaccharide (yield, 2.2%) after ethanol precipitation. With a total sugar content of 80.1%, the molar composition of monosaccharides was reported as 86.8% mannose, 4.5% fucose, 3.9% glucose, 1.6% galactose, 1.2% rhamnose, 1.1% glucuronic acid and 0.9% xylose.

As with small molecular weight drug molecules, the effect of polysaccharides should not be taken as one common pharmacological property. Individual active components must therefore be isolated if one needs to assess the true potential of all polysaccharides in *D. indusiata*. As discussed in the following sections, the activity profile of the crude polysaccharides could be different from the isolated one single polysaccharide. The acid and alkali extracts do also have different compositions with glucose predominant in both cases but Glc, Fru and Man for the acid and Glc and Fru for the alkali extract [22]. Further studies by Hua et al., [6] established their structures and their differential biological activity as evidenced from studies on immunomodulatory and antioxidant effects (Table 2). The exact number of these polysaccharides in the fruiting bodies are still unknown and a simple fractionation based on molecular weight, for example, could yield six components [1].

Overall, the structural feature of *D. indusiata* polysaccharides is predominantly of the *β*-(1→3)-D-glucan with side branches of *β*-(1→6)-glucosyl units that could vary depending on the composition of monosaccharides and their molecular weight. The (1→3)-*α*-D-mannopyranosyl residues that contain *O*-acetyl group do, however, also occur.

### 2.2. Terpenoids

**Figure 3:**
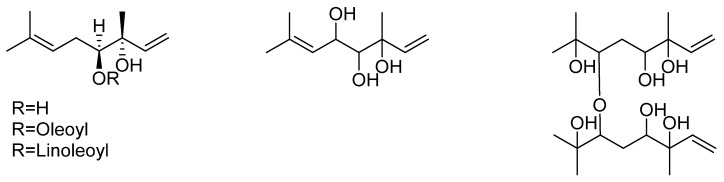
Monoterpenes of *D. indusiata*.

The presence of monoterpenoids in the fruiting body of *D.* *indusiata* was established by the work of Ishiyama et al. [23] that identified five monoterpene alcohols. One of these was (*3R, 4S*)-3, 7-dimethyl-1, 6-octadien-3, 4-diol which was also found as an oleoyl or linoleoyl ester derivative at the 3-hydroxyl position (Figure 3). Other derivatives include a trihydroxyl acyclic monoterpene with undefined stereochemistry and a dimeric ether derivative (Figure 3).

**Figure 4:**
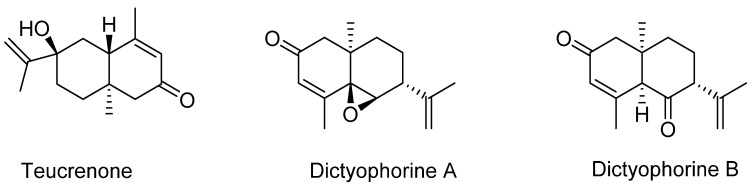
Eudesmane-type sesquiterpenes of *D. indusiata.*

**Figure 5:**
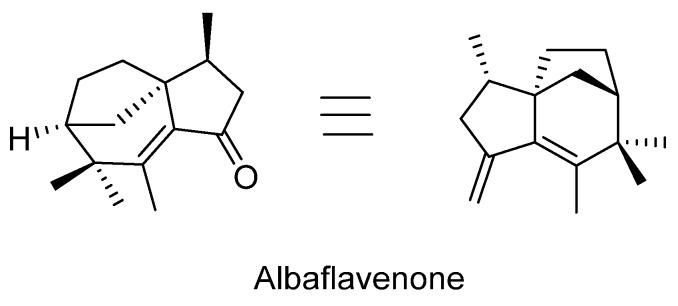
The structure of the tricyclic sesquiterpene antibiotic, albaflavenone.

As a representative of the sesquiterpenes class of terpenoids, Kawagishi et al. [24] have isolated three eudesmane-type compounds, teucrenone and novel derivatives, dictyophorines A and B (Figure 4). The biological significance of these compounds was also shown as they displayed stimulatory effect in astroglial cells to enhance the synthesis of nerve growth factor (NGF). Another representative compound of sesquiterpenes class from the fungus is albaflavenone (Figure 5) which is known for its antibiotic activities. From the dried fruiting body, Huang et al. [25] identified albaflavenone and its concentration was established as about 0.0063% of the dried fungal material.

### 2.3. Alkaloids

**Figure 6:**
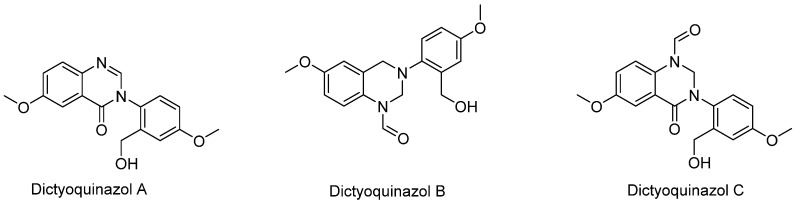
Alkaloids of *D. indusiata.*

The identification of alkaloids from the methanolic extract of *D. indusiata* have been described by Lee et al. [26]. The structures of three of these compounds (dictyoquinazol A–C) are shown in Figure 6. Due to their neuroprotective effect, the synthesis of dictyoquinazol A and derivatives have been studied in recent years with special reference to their potential application in stroke [27]. Oh and Chong Song [28] have also published a six-steps total synthesis procedure for dictyoquinazol A starting from 5-methoxy-2-nitrobenzoic acid in the yield of 36%; while dictyoquinazol B and C were also obtained from dictyoquinazol A as a starting material.

### 2.4. 5-(Hydroxymethyl)-2-Furfural

**Figure 7:**
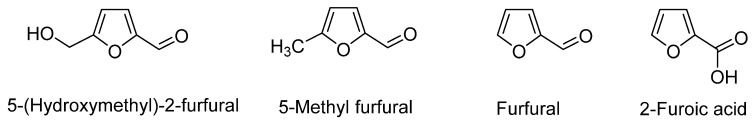
Structures of 5-(hydroxymethyl)-2-furfural *and analogues*.

Sharma et al. [29] identified 5-(hydroxymethyl)-2-furfural (Figure 7) which displayed tyrosinase inhibitory activity. As a natural product, this compound is present in a variety of foods and beverages and its concentration could also rise during drying, storage and heat treatment as an intermediate product of the Maillard reaction. Hence, the non-enzymatic browning of food products that contain sugars and amino acids have been long known to involve the production of 5-(hydroxymethyl)-2-furfural [30,31,32,33]. Not surprisingly, wine and grape juices could accumulate high level of this compound during storage while caramelization (sugar browning) could also yield 5-(hydroxymethyl)-2-furfural. As a natural source, the highest content may be found in roasted coffee while *D. indusiata* is now added to the list of food that contain the compound such as honey, fruit juices and milk, vinegar and beverages. Schematic presentation of 5-(hydroxymethyl)-2-furfural formation from sugars is elegantly presented by Antal et al. [34].

## 3. The Pharmacology of *D. indusiata* Polysaccharides

An overview of in vitro and in vivo effects of extracts and compounds isolated from *D. indusiata* is shown in Table 1 and Table 2. Most of the pharmacological studies are on the polysaccharides that are the major components of the fungus. In the following sections, pharmacological effects with specific mechanism of action and potential therapeutic implications are outlined.

### 3.1. Antioxidant Effect

In an experiment where hydroxyl radical (OH**·**) was generated through iron catalyzed H_2_O_2_ fission, the water-soluble polysaccharides have been shown to display free radical scavenging effect [14,35]. At concentrations far less than 1 mg/mL, the polysaccharides with direct scavenging effect against 1, 1-diphenyl-2-picrylhydrazyl (DPPH), OH**·** and superoxide (O_2_**^−^**) radicals have also been shown [18]. In many experiments, the aqueous extract and crude polysaccharide fractions have been shown to display radical scavenging effects up to the concentration of 2 mg/mL [53]. Consistent with these findings, ABTS^+^ and OH**·**, DPPH, and O_2_**^−^** radicals were suppressed wile lipid peroxidation was inhibited both by the water extract, crude polysaccharides and their sub-fractions [1,46]. The acid-extractable polysaccharides do also have similar effect in scavenging OH·, O_2_**^−^** and DPPH radicals when tested at the concentration range of 0.2–1.4 mg/mL [35]. In addition to similar radical scavenging effects, the reducing power of purified water soluble *β*-d-glucan polysaccharide was further shown [21]. In an attempt to improve the biological activities of the water-insoluble polysaccharides, the sulfated [43] and phosphorylated [13] derivatives have been prepared. In both cases, improvement in the antioxidant activity was observed for the derivatives along with improved water solubility. With the implication of improved bioavailability, it would be interesting to see the in vivo pharmacological activities of these phosphorylated/sulfated derivatives.

In an oxidative hemolysis induced by 2-amidino-propane, a purified polysaccharide considered novel (DP1) has been shown to demonstrate antioxidant and anti-hemolytic effect at exceptionally low dose of 20 nmol/mL. While the level of lipid peroxidation (LPO) marker, malondialdehyde (MDA), and reactive oxygen species (ROS) were suppressed, the activities of intracellular antioxidant enzymes such as superoxide dismutase (SOD), glutathione peroxidase (GPX) and catalase (CAT) were enhanced. Furthermore, the cupric chloride-induced conjugated diene formation in plasma was ameliorated by the DP1 [39]).

The antioxidant activities of polysaccharides have also been demonstrated in animal models. Under parquet-induced oxidative conditions in *Caenorhabditis elegans*, *D. indusiata polysaccharides could* decrease ROS and MDA levels while enhancing SOD activity [19]. It also restored mitochondrial function and integrity as evidenced from, membrane potential and ATP content. The transcription factors SKN-1/Nrf2 and DAF-16/FOXO, which are associated with stress response and lifespan regulation, have been shown to be activated by *D. indusiata* *polysaccharides*. Readers should note that the skn-1 gene in *C. elegans* encodes a transcription factor that resembles the mammalian nuclear factor erythroid 2-related factor 2 (Nrf2). On the other hand, the DAF-16/FOXO transcription in *C. elegans* is the mammalian equivalent of the Forkhead transcription factors with the DNA binding domain or Forkhead box (FOX). This transcription factor is involved in diverse cellular function including the regulation cell death or apoptosis, resistance to oxidative stress and increased life span. Good references for the regulation of these transcription factors are available [54,55,56]. Hence, transcription factors that are critically involved in alleviation of oxidative stress and increased life span of *C. elgans* are activated by *D. indusiata* polysaccharides. In agreement with this data, the acidic and alkali extracted polysaccharides have been shown to increase SOD and GPx activities in the d-galactose-induced senescence in rats [22].

In high fat-induced oxidative damage model, the water soluble polysaccharides administered in mice could abolish the increased LPO (MDA level) while increasing the antioxidant status by elevating SOD, GPx, CAT and total antioxidant capacity (T-AOC) contents/activities in the liver and kidney tissues [14,35]. Similar with this observation was the study by Wang et al. that showed organoprotective (hepatoprotective) effect along with increased antioxidant status in obese and hyperlipidemic mice [48]. Other in vivo model for the demonstration of antioxidant effects was the colitis model in mice where intestinal oxidative stress as assessed by the increased MDA level and GSH depletion markers which were normalized by *β*-glucans [4]. More importantly, the protein expression level of the antioxidant hem oxygenase-1 (HO-1) was raised in colonic tissues. This is consistent with another study on colitis model in mice where the crude polysaccharides have been shown to enhance the level of SOD activities while lowering nitric oxide (NO) level in colonic tissues [49]. All these data reveal the potential therapeutic implication of *D. indusiata* polysaccharides in pathologies associated with ROS and/or oxidative stress.

### 3.2. Neuroprotective Effect and Potential Application in Neurodegenerative Diseases

By using wild-type and transgenic *C. elegans* models, Zhang et al. [19] studied the neuroprotective effect of *D. indusiata* polysaccharides under various conditions. In addition to the above-mentioned antioxidant effects (see Section 3.1), the acidic polysaccharides have been shown to mitigate the polyglutamine and amyloid-*β* protein-induced chemosensory behavior dysfunction in transgenic nematode models of neurodegenerative disease. Hence, the antioxidant mechanism including the Nrf2 pathway and inhibition of the polyglutamine- and A*β*-mediated neurotoxicity suggest the potential therapeutic application of *D. indusiata* for neurodegenerative diseases. The reversal of oxidative stress by acidic and alkali extracted polysaccharides of *D. indusiata* in d-galactose-induced senescence in mice [22] also imply potential amelioration of age-related disorders such as Alzheimer’s disease.

The small molecular weight compounds of *D. indusiata* have not yet been extensively studied for their neuroprotective effects. In the study by Kawagishi et al. [24], the eudesmane-type sesquiterpenes isolated from the aqueous alcohol (74% ethanol) extract were assessed for their potential effect on astroglial cells. They have shown that they could enhance the synthesis and subsequent release of NGF by four-fold when the cells were treated by dictyophorine A (3.3 μM). Since dictyophorine B was active, but with less potency, the epoxy functional group in these eudesmane-type compounds (Figure 4) appears to be an important structural feature for NGF release. The role of NGF and agents that promote its release and function as potential therapeutic agents for Alzheimer’s disease and related neurodegenerative diseases have been extensively reviewed [57]. On the basis of the in vitro data, more research on the mechanisms and in vivo effect of dictyophorine A, or *D. indusiata* extracts as neuroprotective agents, is therefore well justified.

In their pioneering work, Lee et al. [26] were the first to characterize and establish the neuroprotective effect of dictyoquinazols that they isolated and characterize from *D. indusiata*. The compounds (dictyoquinazol A, B, and C, Figure 6) were able to protect primary mouse cortical neurons from excitotoxicity and cell death induced by glutamate and NMDA. More importantly, the dose-dependent effect was evident in the lower μM concentration range (up to 5 µM).

### 3.3. Anticancer Effect

The direct cytotoxic effects of the polysaccharides against cancer cells may be seen as generally weak and occur at higher μM concentration at best. The triple helical polysaccharide (PD3), for example, did not display cytotoxicity against mouse sarcoma S180 cells in vitro when tested up to 1 mg/mL though it displayed impressive effect in vivo [15]. In contradiction to this observation, however, the crude polysaccharide has been shown to display direct cytotoxicity in the same cell line, osteosarcoma S180 cells [45]. This effect was also seen at moderate concentration range of 10–160 μg/mL. The dose-dependent effect was also in line with induction of apoptosis as evidenced from morphological, biochemical and gene expression analysis (Table 1). Other studies also showed the direct cytotoxic effects of the water extract and crude polysaccharides against HeLa and HepG2 cells within the concentration range of 100–600 μg/mL [46].

To enhance the cytotoxic activity of the purified polysaccharide, DP1, Liao et al. [41] also prepared a zinc chelate product. The direct cytotoxicity of this derivative against MCF-7 cells was shown through induction of apoptosis as evidenced through the classical DNA fragmentation, cell cycle arrest (S-phase) and activation of caspases (caspases-3, -8, and -9). A further possible mechanism for the induction of apoptosis was mitochondrial dysfunction and ROS overproduction induced by the zinc chelate of DP1. Induction of ROS overproduction through the mitochondrial respiratory pathways leading to caspases activation and apoptosis appears to be consistent to the anticancer activity of many natural products [58]. The same group also prepared a monodispersed selenium nanoparticle of DP1 that induce apoptosis in HepG2 cells through exactly the same above-mentioned mechanism [40]. Other derivatization studies were based on phosphorylated and sulfated products which showed more cytotoxic effect against MCF-7 and B16 cells than the parent polysaccharides [13,43]. A further advantage of this approach lies on the water solubility of the sulfated/phosphorylated derivatives as compared to the water insoluble starting material which may have relevance to in vivo applications.

Several animal experiments have also been employed to demonstrate the potential anticancer effect of *D. indusiata* polysaccharides. In S180 tumor bearing mice, the triple helical polysaccharide (PD3) administered intraperitoneally (i.p.) for ten days has been shown to suppress tumor size and reverse body weight loss [15]. This could be partly due to the immunostimulant effect of the polysaccharide (see Section 3.4). Both *β*-(1→3)-D-glucans and *α*-(1→3) linked D-mannan polysaccharides from *D. indusiata* have also been shown to suppress tumor growth in S180 tumor bearing mice [51]. The maximum doses employed as 25 mg/kg via the i.p. route is also encouraging for further studies in this field.

### 3.4. Immunomodulatory Effect

Readers should make the distinction between therapeutic application via an immunestimulatory effect and immunosuppression. Polysaccharides could do both, and while immunostimulation is applicable when the immune system is suppressed such under cancer pathology, immunosuppression is applicable under chronic inflammatory conditions such as colitis, sepsis, etc. The effect of *D. indusiata* compounds described in the following sections under these two contrasting conditions should not therefore be seen as a contradiction.

#### 3.4.1. Immunostimulation

The immunestimulatory effect of *D. indusiata* polysaccharides has been demonstrated in unstimulated macrophages in vitro. Treatment of the murine RAW264.7 cells with these polysaccharides could induce a proliferative response while markers of macrophage activation such as cytokines (IL-1*β*, IL-6 and TNF-*α*), NO synthase (level of NO) and *nuclear factor* κ-light-chain-enhancer of activated B cells (NF-κB) p65 were upregulated [38]. Moreover, macrophage activation by these polysaccharides could be attributed to the toll-like receptor 4 (TLR4) since the observed activity could be abolished by anti-TLR4 and anti-CR3 mAbs. In another study by the same authors, one purified polysaccharide (Dectin-1) was shown to induce other markers of macrophage activation in RAW264.7 cells including pseudopodia formation, cell spreading and phagocytosis [20]. In addition to the above-mentioned specific binding to TLR4, cytokine (IL-1*β* and TNF-*α*) expression, and NF-κB activation and nuclear translocation, Dectin-1 has been shown to induce the phosphorylation of the extracellular signal-regulated kinase 1/2 (ERK1/2), JNK1/2 and p38 mitogen-activated protein kinase (MAPK) [20]. As part of the immunostimulation process, macrophage activation with specific recruitment of M1-phenotype leading to tumour suppression has been shown to be a function of these MAPKs activation [59]). It has also been shown that the immunestimulatory effect of polysaccharides like that obtained from *Laminaria japonica* is through TLR4 as a recognition receptor leading to ERK1/2, JNK1/2 and P38 activation, NF-κB p65 mobilization from the cytoplasm to nucleus, and cytokines and NO overproduction in macrophages [60].

The study by Fu et al. [44] on immunestimulatory effect of the polysaccharides were similar with the above studies by Deng et al. [20,38]. They have shown however that the purified polysaccharide of *β*-(1→3)-glucan with side branches of *β*-(1→6)-glucosyl unit had a triple-helical structure. It promoted the proliferation of RAW 264.7 macrophage along with induction of NO and cytokine (TNF-*α*, IL-1, IL-6, and IL-12) production. Animal experiments using the acid and alkali extractible polysaccharides also showed increased macrophage phagocytosis power and NK cells killing activity (only for the alkali extract) [44].

A classic example of immnunostimulation and a further potential application of *D. indusiata* polysaccharides to cancer therapy was highlighted by the study of Han et al. [37] which employed the supernatant of prostate cancer fibroblasts to suppress immune cells. Under this condition, they have shown that the polysaccharides could stimulate lymphocyte proliferation and could ameliorate the suppressed growth of CD4+/CD8+ T cells. As a direct link between cancer and immunosuppression, the potential effect of triple helical polysaccharide (PD3) was also assessed in ascitogenous sarcoma S180 bearing mice [15]. The tumor suppressive effect of this polysaccharide was shown to be associated with increased level of cytokines in the blood such as IL-2, IL-6, and TNF-*α*.

#### 3.4.2. General Anti-Inflammatory, Immunosuppressive and Effect on the Gut Microbiota

Wang et al. [36] employed the classical LPS-stimulated macrophage activation assay to assess the anti-inflammatory potential of *D. indusiata* polysaccharides. At fairly small doses (25–50 μg/mL), the LPS-induced cytokine (IL-1) and ROS production, as well as TLR4 expression and NF-κB activation/nuclear translocation, were suppressed. By using the carrageenan-induced oedema model of the classical inflammation model and scalded edematous hyperalgesia in rats’ hind paws, Hara et al. [52] were the first to demonstrate the ant-inflammatory potential of *D. indusiata β*-glucans.

One of the best anti-inflammatory activity studies for the *D. indusiata β*-glucans was based on the dextra sulphate sodium (DSS)-induced colitis model in mice [4]. In this study, oral administration of up to the maximum doses of only 100 mg/kg effectively reversed colonic length and inflammatory and oxidative markers (Table 2). Of the inflammatory markers suppressed were cytokines’ gene expression for TNF-*α*, IL-6, IL-1*β* and IL-18 and myeloperoxidase (MPO) activity. At the biochemical level, apoptosis markers were suppressed, tight junction proteins (TJP-1 protein expression) were abundant, the expressions for nucleotide-binding domain leucine-rich repeats family protein 3 (NLRP3), phosphorylated signal transducer and activator of transcription 3 ((p)-STAT3) and p-IκB*α* (phosphorylated nuclear factor of kappa light polypeptide gene enhancer in B-cells inhibitor, alpha) enhanced, and M1 macrophage (F4/80^+^CD11b^+^ cells) polarization were down-regulated while the M2 (F4/80^+^CD206^+^ cells) subsets in splenic tissues were raised. Another study using the DSS-induced colitis model in mice was that employed by Kanwal et al. [49] where the crude polysaccharide at a maximum oral dose of only 33 mg/kg was tested. In addition to amelioration of the physical markers of colitis (Table 2), the enhanced mucins and tight junction proteins expressions along with improved antioxidant status were evident. As an anti-inflammatory agent, the crude polysaccharides also suppressed the levels of proinflammatory cytokines and MPO activity, boost the level of anti-inflammatory cytokines, and ameliorate the expression of iNOS and NFκB activation.

In addition to the above-mentioned anti-inflammatory properties of *D. indusiata* polysaccharides in the ulcerative colitis model demonstrated by Wang et al. [4], the decrease in the abundance of *Firmicutes* and increased *Proteobacteria* levels under a similar DSS-induced colitis model in mice were reversed by the administration of the crude polysaccharides [49]. Another comprehensive study on the effect of *D. indusiata* polysaccharides on gut microbiota came from the study by Kanwal et al. [7]. Under broad-spectrum antibiotic-mediated dysbiosis and intestinal barrier dysfunction, *D. indusiata* polysaccharides have been shown to recover the altered (Firmicutes/Bacteroidetes ratio and increased the relative abundance of harmful flora such as Proteobacteria, *Enterococcus*, and *Bacteroides*) gut microbiota composition. More specifically, they have reported that the polysaccharides enhance the level of beneficial flora, such as Lactobacillaceae (lactic acid-producing bacteria), and Ruminococaceae (butyrate-producing bacteria). Moreover, the lipopolysaccharides were shown to suppress endotoxemia and the level of pro-inflammatory cytokine TNF-*α*, IL-6, and IL-1*β*, while the expression of tight-junction associated proteins (claudin-1, occludin, and zonula occludens-1) were increased. All these anti-inflammatory effects coupled with improvement of the gut microbiota and restoration of tight junction and mucus barriers demonstrated by Kanwal et al. [49] imply the potential immunomodulatory effect of *D. indusiata* polysaccharides.

### 3.5. Antiobesity and Potential Antidiabetic Effect

The comprehensive antiobesity potential study by Wang et al. [14,35] employed a high fat-induced obesity model where the water-extractible polysaccharides of *D. indusiata* were administered in mice orally for 45 days. As shown in Table 2, the obesity-induced hypercholesterolemia, increased liver enzyme markers in the serum, and liver and kidney morphological changes and oxidative stress were ameliorated. These activities imply a wider effect of organoprotective effect primarily where oxidative stress is implicated and also in obesity-associated diseases like diabetes and cardiovascular complications. Wang et al. [48] also employed the high-fat-induced obesity and hyperlipidemia model to assess the potential antidiabetic effects of *D. indusiata* polysaccharides obtained by enzyme assisted or alkali extraction method (Table 2). They have demonstrated a direct glucose lowering effects under OGTT while the alteration in the serum adiponectin level and the increases in insulin and leptin were normalized. All these data [47] were in addition to the antioxidant, organoprotective (hepatocytes), antiobesity (weight gain reduction) and antihyperlipidemic effect (Table 2), which were also reported previously [14,35]. As the obesity associated alteration in the level of urea, creatinine, albumin, insulin, leptin and adiponectin were reversed by the polysaccharides [14,35,47], a further comprehensive study on the antidiabetic potential of *D. indusiata* polysaccharides is well merited.

### 3.6. Antibacterial Effects

Chen et al. [25] described the isolation and characterization of the antibiotic albaflavenone from *D. indusiata* which has been widely known for its antibacterial activities. As a component of Streptomyces (e.g., *Streptomyces albidoflavus, S. coelicolor*), its biosynthesis pathway and activities have been the subject of many studies [61,62,63,64]. As part of the antioxidant and antimicrobial activity study, Oyetayo et al. [53] also reported that the water extract of *D. indusiata* can inhibit the growth of bacteria and fungi. The reported activity was however not of therapeutic relevance and was 200 mg/mL. Since the known antibiotic principle is a lipophilic terpenoid, albaflavenone, the poor antimicrobial activity of the water extract only suggests the lack of water soluble antibiotic agent in the fungi.

### 3.7. Other Effects

5-(hydroxymethyl)-2-furfural isolated from the methanolic extract of *D. indusiata* has been shown to display tyrosinase inhibitory effect in a non-competitive manner [29]. In view of the functional role of tyrosinase enzyme in melanogenesis, insect metabolism and fruits and vegetables spoilage due to browning and taste change, its inhibitors have long been appreciated in the medical, cosmetics and agrochemical industries. Numerous natural products such as phenolics and the gold standard reference compound, kojic acid, are known for their tyrosinase inhibition. Sharma et al. [29] also screened three analogues of 5-(hydroxymethyl)-2-furfural (Figure 7) for their tyrosinase inhibitory effect. Furfural (IC_50,_ 0.35 mM) but not 2-furoic acid was active suggesting the role of the aldehyde functional group for the observed activity. On the other hand, 5-methyl furfural (IC_50,_ 0. 76 mM) had comparable inhibitory effect as 5-(hydroxymethyl)-2-furfural (IC_50,_ 0.98 mM). We should note however that this level of potency and the non-competitive nature of inhibition do not make 5-(hydroxymethyl)-2-furfural as a significant lead compound for the discovery of novel tyrosinase inhibitors as depigmentation agents in medicine/cosmetics or anti-browning agents in the food/agricultural industries.

Wang and Ng [65] isolated a 28 kDa ribonuclease (RNase) with specific activity of 564 U/mg towards yeast transfer RNA. The potential effect of the RNase in other fields need to be assessed given other RNase from mushrooms such as *Hohenbuehelia serotina* have been shown to inhibit the human immunodeficiency virus type 1 (HIV-1) reverse transcriptase and inhibit leukemia (L1210) cells and lymphoma (MBL2) cells proliferation [66].

### 3.8. Toxicity Remarks

*D. indusiata* is a common food particularly in the far eastern countries such as China and Japan. Its toxicity is thus not of a concern unless excessive amounts of, for example, extracts, and isolated compounds are consumed. The water-soluble polysaccharides [14] and those obtained by alkali and enzyme-assisted extraction [47] have been routinely tested for acute toxicity in mice. Oral administration of the extract at the oral dose of up to 1200 mg/kg for two months was not reported to exhibit any behavioral changes and toxic symptoms. While similar reports on the animals tested on were common for the indicated doses in Table 2, there has been no report on toxicological concern for *D. indusiata.*

### 3.9. General Summary and Conclusion

In comparison to plants, mushrooms are the growing but yet underexploited natural resources both as food and as sources of valuable medicines. In recent years, considerable attention has been given to higher basidiomycete which are considered as medicinal mushrooms based on their nutritional value and therapeutic implications. The significance of bioactive molecules from such mushrooms like the *Agaricus, Auricularia, Ganoderma, Lentinus*, *Phellinus, Pleurotus* and *Trametes* genera have been reviewed [67]. One advantage of sourcing bioactive compounds from mushrooms is the inherent ability to cultivate them not just in the field but also under submerged culture condition in bioreactors. In comparison to the medicinal mushrooms of the higher basidiomycete, the chemical and pharmacological studies on *D. indusiata* only begun in earnest during the 1980s. Interestingly, the major components and biologically active compounds in this fungus are the carbohydrate class like those considered as the bioactive principles in the genus *Ganoderma* and *Trametes*. The plethora of evidences presented in the scientific literature for polysaccharides of these higher basidiomycete fungi as antioxidant, organoprotective, anticancer, immunomodulatory, antidiabetic/antiobesity, etc. are thus in good agreement with the assertion of therapeutic potential outlined in this communication for *D. indusiata.* Although mushrooms contain a wide range of polysaccharides such as chitin, mannans, galactans, and xylans, the prevalent bioactive polysaccharides in the higher medicinal mushrooms is *β*-glucans of the *β*-(1→3) linkages with some *β*-(1→6) branches [68,69,70]*. In this context, studies on the physicochemical characterization of* *D. indusiata* polysaccharides so far has revealed important structural information on the general structural backbones of the bioactive compounds although more work is needed to isolate and characterize all the individual polysaccharides. In comparison to the polysaccharides, studies on the small molecular weight bioactive compounds of *D. indusiata* is still in its infant stage. Although, some terpenoids and alkaloids have been characterized with limited pharmacological effects, the lipophilic or small molecular weight secondary metabolites that could be manipulated in culture by biotechnology tools remains to be investigated.

Although a direct radical or ROS scavenging effect is a function of the structurally optimized phenolic compounds such as gallates, phenyl propanoids and flavonoids, the antioxidant effects of polysaccharides appear to represent the most studied biological activity of medicinal mushrooms. As expected, the direct radical/ROS scavenging effect of *D. indusiata* polysaccharides is moderate at best. In recent years however, compounds that are not optimized for direct ROS scavenging have been shown to display antioxidant effects in vivo owing to their ability to boost antioxidant defenses such as SOD, GPx, and CAT. In this connection, an emerging mechanism of action for such natural products is the redox sensitive transcription factor Nrf2. Hence, compounds with weak radical scavenging effects can induce considerable antioxidant effects in vivo by upregulating the expression of Nrf2 leading to the activation of antioxidant genes and proteins including glutamate-cysteine ligase, glutathione peroxidase 1, thioredoxin reductase 1, NAD(P)H-quinone oxidoreductase 1, glutathione-*S*-transferase, SOD, CAT, peroxiredoxin, ferritin and HO-1 [71,72,73]. The demonstration of *D. indusiata* polysaccharides to increase this antioxidant status under various experimental conditions in vitro and in vivo suggest their wider potential as antioxidant agents. Since oxidative stress is associated with major pathologies such as cancer, inflammation, neurodegeneration, cardiovascular diseases, etc., the demonstration of potential application of *D. indusiata* polysaccharides in the various experimental models could also be an attribute of antioxidant mechanism of action.

By boosting the level of expression of proinflammatory cytokines such as IL-2, IL-6, and TNF-*α*; enhancing macrophage polarization and activity, and direct anticancer effect in vitro and in vivo, *D. indusiata* polysaccharides appear to display potential as anticancer agents through two distinct mechanism of action: direct antitumor effect and immunostimulation. This is in line with other medicinal mushroom polysaccharides such as those from *Coriolus versicoloris* [74,75]. The observed mechanism of action including NF-κB p65 activation and mobilization to the nucleus, phosphorylation and activation of the ERK1/2, JNK1/2 and p38 MAPKs and the involvement of TLR-4 as a recognition receptor for *D. indusiata* polysaccharides is also in line with what has been established for *C. versicolor* polysaccharides [76]. On the other hand, the anti-inflammatory effect *D. indusiata* polysaccharides have been established in a variety of experimental conditions both in vitro and in vivo. Hence, under pathological conditions such as LPS-induced macrophage activation and endotoxemia and experimentally induced gut inflammation such as colitis and antibiotic damage or neurodegenerative models, the anti-inflammatory effect of these polysaccharides are evident.

In recent years, there has been a growing understanding that alteration in the composition of the gut microbiota is a major risk to the development of many diseases. Therapeutic approaches to normalize the Bacteroidetes and Firmicutes composition have thus been employed to improve food digestion/bioavailability, improve the immune response and protect the barrier function of the gut, and even to modify the metabolism of drugs and xenobiotics. The prebiotic nature of mushroom polysaccharides as a mechanism for their therapeutic effects and how they alter the intestinal microbiota composition have been extensively researched [77,78]. In line with these observations, the polysaccharides of *D. indusiata* appear to ameliorate intestinal damage and inflammation while increasing the proportion of the gut microbiota associated with good health (e.g., *Lactobacillus spp*.) and reducing the composition of those linked to pathologies.

The antiobesity and antidiabetic potential study on *D. indusiata* is just beginning to take shape and the available data so far is encouraging to do more compressive research in this field. The studies by Wang et al. [14,35,48] were particularly impressive where antiobesity and antihyperlipidemic effects were observed in obese mice. The classical atherogenic index and lipid markers were lowered while biochemical and histopathological markers of liver and kidney damage were reversed along with amelioration of oxidative stress markers. Furthermore, preliminary studies on the potential antidiabetic effect was observed from the reduction of blood glucose under OGTT [47]. A more comprehensive study in this area is however required though the trend is similar with the antiobesity, antihyperlipidemic and antidiabetic effects of polysaccharides isolated from higher fungi like *Ganoderma lucidum* [79,80,81]. Associated with obesity and diabetes pathology is also the altered gut microbiota composition which other mushroom polysaccharides like those from *G. lucidum* have also been shown normalize [82,83].

Overall, studies both on the chemistry and pharmacology of *D. indusiata* extracts and compounds are increasing in recent years and show therapeutic potential for various pathologies. Since the mushroom is considered as non-toxic with a significant volume of consumption especially in the Far East, more comprehensive studies on the small molecular weight constituents and polysaccharides are well merited. One other area of interest is the diversification of research to maximize the accessibility of the bioactive compounds and their yield through biotechnology tools.

## Figures and Tables

**Table 1:** Evidences of pharmacological efficacy from in vitro studies.

Preparation	Experimental Model	Key Findings	References
Water-extractable polysaccharides	Antioxidant assay—0.2–1.0 mg/mL	Scavenge DPPH and OH^.^ radicals; possess reducing power.	Wang et al. [14]
Acid-extractable polysaccharides -average molecular weight of estimated to be 7.89 × 10^5^ Da, 4.64 × 10^5^ Da and 6.41 × 10^5^ Da	Antioxidant assay—0.2–1.4 mg/mL	OH^,^ O_2_^−^ and DPPH radical scavenging effect.	Wang et al. [35]
Polysaccharides	LPS-stimulated RAW264.7 macrophages—25 μg/mL or 50 μg/mL	Inhibit the expression of TLR4, phosphorylated-IκB*α* and NF-κB nuclear translocation (p65 subunit); decrease the extracellular protein level of pro-caspase-1 and cytoplasmic pool of NLRP3; inhibit the level of activated caspase-1, and caspase-1-mediated IL-1 and IL-18 production; inhibit IL-1 and ROS production.	Wang et al. [36]
Purified polysaccharide—Dectin-1	RAW264.7 cells—200 μg/mL	Induce pseudopodia formation and cell spreading; increase phagocytic uptake; enhance IL-1*β* and TNF-*α*; induce phosphorylation of ERK1/2, JNK and p38; enhance the phosphorylation of NF-κB p65 and NF-κB nuclear translocation; specifically bind to macrophages through TLR4.	Deng et al. [20]
Polysaccharides	Radical scavenging assay	EC_50_ for DPPH, OH^.^ and O_2_^−^ was 0.89 mg/mL, 0.51 mg/mL and 0.68 mg/mL respectively.	Liu et al. [18]
Polysaccharides	Immunosuppressive effect of prostate-cancer-associated fibroblasts—supernatant of prostate cancer fibroblasts on lymphocyte growth—0.1–0.8 mg/mL	Stimulate the proliferation of immune cells and reverse inhibition of the growth of CD4+/CD8+ T cells; down-regulate the expression of *α*-smooth muscle actin.	Han et al. [37]
Polysaccharides	RAW264.7 cells—25–200 μg/mL	Proliferative effect, up-regulate the production of NO, IL-1*β*, IL-6 and TNF-*α* (both protein and m RNA expression); effect mediated by TLR4 (inhibited by anti-TLR4 and anti-CR3 mAb); specifically bind to macrophage in TLR4-dependent manner; activate NF-κB p65.	Deng et al. [38]
Purified polysaccharide (DP1)	2, 2-azobis (2-amidino-propane) dihydrochloride -induced erythrocyte hemolysis assay—20 nmol/mL	Inhibit hemolytic activity by 87.4%; inhibit ROS overproduction by 81.5%; suppress MDA level by 57.0%; inhibit the cupric chloride- induced conjugated diene formation in plasma; enhance intracellular antioxidant enzymes (SOD, GPX and CAT) activities.	Liao et al. [39]
Purified polysaccharide (DP1)—monodispersed selenium nanoparticles	HepG2 cells—125, 250 or 500 μg/mL	Induce cell apoptosis—nuclear condensation, DNA cleavage and accumulation of S-phase cell arrest; activate caspases 3, 8 and 9; induce ROS overproduction and mitochondrial dysfunction.	Liao et al. [40]
Purified polysaccharide (DP1) chelated with zinc chloride	MCF-7 cells—125 or 250 μg/mL	Induce apoptosis—DNA breakage, and S-phase cell cycle arrest, activate caspases-3, -8, and -9, mitochondrial dysfunction, and ROS overproduction.	Liao et al. [41]
Polysaccharide—an average molecular weight of 1132 kDa and consisted of glucose (56.2%), galactose (14.1%), and mannose (29.7%). The main linkage type of DP1 were proven to be (1→3)-linked *α*-L-Man, (1→2, 6)-linked *α*-D-Glc, (1→6)-linked *β*-D-Glc, (1→6)-linked *β*-D-Gal, and (1→6)-linked *β*-D-Man	RAW 264.7 cells—up to 250 μg/mL	Promote macrophage NO, TNF-*α*, and IL-6 secretion.	Liao et al. [42]
Phosphorylated polysaccharides prepared from a water-insoluble polysaccharides	Radical scavenging assay; cytotoxic effects against MCF-7 and B16 cells	Comparative study—phosphorylated derivative more potent than native polysaccharides.	Deng et al. [13]
Water-soluble sulfated polysaccharides—prepared from water insoluble polysaccharides	OH^.^ and DPPH radical scavenging assay; cytotoxicity against MCF-7 and B16 cells	Comparative study—Sulfated derivative more potent than native polysaccharides.	Deng et al. [43]
Water-soluble polysaccharide of *β*-(1→3)-glucan with side branches of *β*-(1→6)-glucosyl unit—triple-helical structure	RAW 264.7 macrophage—50–200 µg/mL	Promote macrophage cell proliferation; increase NO and cytokines (TNF-*α*, IL-1, IL-6, and IL-12) production.	Fu et al. [44]
Crude polysaccharides	Cytotoxicity against osteosarcoma S180 cells—10–160 µg/mL	Induce cell death and DNA fragmentation; modulate the expression of apoptosis associated genes and proteins (increase bcl-2 and decrease cdk4 and p53); activate caspase-3.	Zhong et al. [45]
A triple helical polysaccharide (PD3)	Mouse sarcoma S180 cells—0.2, 0.5 or 1 mg/mL	No direct cytotoxicity against S-180 cells.	Deng et al. [15]
Water-soluble polysaccharide—purified by gel chromatography (Sephadex G-200)—*β*-glucan mainly consist of glucose (98.58%).	Antioxidant assay—25–1000 µg/mL	Display reducing power; OH^.^, O_2_^−^ and DPPH radical scavenging activity.	Deng et al. [21]
Water extract and crude polysaccharides:	Antioxidant assay; HeLa and HepG2 cells —100–600 µg/ml	Scavenge ABTS^+^ and OH^·^ radicals; inhibit lipid peroxidation; inhibit cancer cell proliferation.	Li et al. [46]
Six polysaccharides sub fractions with molecular weight range of 801–4656 kDa.	Antioxidant assay—1–5 mg/mL	The smallest MW 801 kDa, exhibit the most potent scavenging effect against the DPPH, OH^.^, and O_2_^−^ radicals—IC_50_ values 0.11, 1.02 and 0.64 mg/mL respectively.	Ker et al. [1]
Dictyoquinazols A, B, and C	Primary cultured mouse cortical neurons—5 µM	Protect from glutamate- and NMDA-induced excitotoxicity.	Lee et al. [26]
Dictyophorines A and B, and a known compound, teucrenone	Astroglial cells—5 µM	Stimulate NGF synthesis and release.	Kawagishi et al. [24]
Homogeneous polysaccharides and a conjugated polysaccharide fraction—Fucomammogalactan (T-3-Ad), conjugated polysaccharide fraction (T-2-A), *β*-(1→6)-branched -(1→3)-*β*-D-glucans (T-4-N and T-5-N); Partially *O*-acetylated 1→3)-*α*-D-mannans (T-2-HN and T-3-M’).	C3H/He spleen cells—concentration expressed as 10 µg/well	T-3-Ad and T-2-A exhibit significant mitogenic and CSF-inducing activities; T-4-N but not T-5-N showed both mitogenic and CSF-inducing effects. T-2-HN and T-3-M did not show activity.	Hara et al. [47]

Abbreviations: ABTS^+^, 2,2’-azino-bis(3-ethylbenzothiazoline-6-sulfonic acid) cation; bcl-2, B-cell lymphoma 2; CAT, catalase; cdk4, *cyclin-dependent kinase 4*; DPPH, 1,1-diphenyl-*β*-picrylhydrazyl; ERK1/2, extracellular signal–regulated kinase-1/2; FOXO, Forkhead box protein O; Fru, fructose; Gal, galactose; Glc, glucose; GSH, glutathione; GPx, glutathione peroxidase; IFN, interferon; IκB, inhibitor of κ-B; IL- interleukin; JNK, c-Jun N-terminal kinase; LPS, lipopolysaccharide; mAb, monoclonal antibody; Man, mannose; MDA, malondialdehyde; NF-κB, *nuclear factor* κ-light-chain-enhancer of activated B cells; NGF, nerve growth factor; NMDA, N-Methyl-d-aspartic acid or N-Methyl-d-aspartate; NLRP3, nod-like receptor family pyrin domain containing 3; NO, nitric oxide; OH**^.^**, Hydroxyl radical; O_2_**^−^** superoxide radical; ROS, reactive oxygen species; SOD, superoxide dismutase; TTLR4, Toll like receptor-4; TNF, tumor necrosis factor.

**Table 2:** Evidences of pharmacological efficacy from in vivo studies.

Preparation	Experimental Model	Key Findings	References
Water-extractable polysaccharides	High fat-induced obesity in mice—400 mg/kg, p.o. for 45 days	Reduce the serum level of TC, TG and LDL-C while enhancing HDL-C level; suppress the obesity-induced raised activities of ALT, AST, ALP, LDH and CK enzymes; improve the liver and renal antioxidant status (increase SOD, GPx, CAT and T-AOC contents/activities and suppress MDA, LPO and MPO contents; reduce hepatic lipid levels (TC, TG and NEFA); reverses the obesity associated increase in urea and creatinine or reduced albumin; ameliorate the obesity associated increased insulin and leptin and suppressed adiponectin level; restored morphological changes of the kidney and liver (histopathological study).	Wang et al. [14]
Acid-extractable polysaccharides -average molecular weight of estimated to be 7.89 × 10^5^ Da, 4.64 × 10^5^ Da and 6.41 × 10^5^ Da	High fat-induced obesity in mice—400 mg/kg, p.o. for 45 days	Data similar as above (Wang et al., 2019a [1])	Wang et al. [35]
Alkali (NaOH solution as 0.5 mol/L, 1:10, *w/v* at 85 °C for 5 h) or enzyme (snailase solution as 4%, 1:4, *w*/*v*) at 38 °C for 4 h) extractable polysaccharides	High fat-induced hyperlipidemia and obesity in mice—100 or 400 mg/kg, p.o. for 33 days	Reduce body weight gain; decrease the serum levels of TC, TG, LDL-C and atherogenic index; increase HDL-C in serum; improve hepatic lipid levels (TC, TG and NEFA); decrease serum enzyme activities levels of liver toxicity marker enzymes (ALT, AST, ALP, LDH, CK and TBIL; reverse the decreased antioxidant enzyme activities (SOD, GPx and CAT), reduce non-enzymatic antioxidant capacity (T-AOC), as well as increased lipid product contents (MDA and LPO); improve hepatocyte morphology (histopathological observation); reverse the hyperlipidemia-induced decrease in adiponectin level and the increases in insulin and leptin in serum; lower improve the increased blood glucose level under the OGTT.	Wang et al. [48]
Polysaccharides—homogeneous *β*-(1→3)-D-glucan with side branches of *β*-(1→6)-glucosyl units	DSS-induced colitis in mice—25, 50 or 100 mg/kg p.o. for 7 days	Attenuate colitis severity (colonic length and macroscopic features, tissue architecture and inflammation score); reduce splenomegaly; suppress intestinal oxidative stress (suppress MDA while increasing GSH level; enhance HO-1 protein expression level); suppress the expression of inflammatory cytokines (TNF-*α*, IL-6 and IL-1*β*, IL-18 gene expression) and MPO activity in colonic tissues; abolish the induction of protein expressions for NLRP3, phosphorylated (p)-STAT3 and p-IκB*α*; ameliorate the induce apoptosis and deletion (increased Bcl2 while reducing Bax protein expressions) of tight junction protein (TJP-1 protein expression) in colonic tissues; down-regulate M1 macrophage (F4/80^+^CD11b^+^ cells) polarization or increase he M2 (F4/80^+^CD206^+^ cells) subsets in splenic tissues.	Wang et al. [4]
Crude polysaccharides—total sugar content was 96.66% with 13.2% polysaccharide yield. Glc 59.84%, Man 23.55% and Gal 12.95%	DSS-induced colitis in mice—10 or 33 mg/kg, p.o. for two weeks before and during the DSS	Abolish clinical symptoms—recovery of body weight loss and disease markers (stool consistency and rectal bleeding, colon length, inflammatory score); improve colonic histological changes; enhance mucins and tight junction proteins (increased expression level of claudin-1, occludin, and zonula occludins (ZO-1, and ZO-2)) expression (assessed by histopathological studies); reduce the MPO and NO; enhance SOD levels in colonic mucosa; reduce the production of pro-inflammatory cytokines (TNF-*α*, IL-1*β*, IL-6, IFN-γ) and IL-17 while enhancing the anti-inflammatory cytokines (IL-4, IL-10) in colonic mucosa; decrease the phosphorylation of p65, IκB-*α* and ERK; suppress the expression of iNOS; enhance alpha diversity indices; reverse the dysbiosis pattern—the DSS-induced decrease in abundance of *Firmicutes* and increase *Proteobacteria* bacteria.	Kanwal et al. [49]
Crude polysaccharide	Dysbiosis-induced by broad-spectrum antibiotics (clindamycin metronidazole) in BALB/c mice—0.2 mg/0.2 mL) p.o.	Restore body weight loss; restore the reduced bacterial diversity—increase Lactobacillaceae, Ruminococaceae, S24-7, and Odoribacteraceae while reducing Bacteroidaceae, Enterococcaceae), and Enterobacteriaceae; ameliorate colon wall damage and inflammation; downregulate TNF-*α*, IL-1*β*, and IL-6 (protein levels); reduce endotoxins; increase the expression of tight-junction proteins (claudin, occludin, and ZO-1); restore mucus layer thickness (modulate mucin-2 protein expression).	Kanwal et al. [7]
Acidic polysaccharide isolated by anion-exchange chromatography	Wild-type *C. elegans* under parquet-induced oxidative stress and transgenic *C. elegans* of neuroprotection—1.0 and 2.0 mg/mL	Decrease ROS and MDA levels and increase SOD activity; restore the functional parameters of mitochondria, (membrane potential and ATP content) in parquet-stressed nematodes; effect dependent on stress response transcription factor DAF-16/FOXO; reduce ROS levels and alleviate chemosensory behavior dysfunction in transgenic *C. elegans* mediated by polyglutamine and amyloid-*β* protein.	Zhang et al. [19]
A triple helical polysaccharide (PD3)	Tumor cell suspension from ascitogenous sarcoma S180 mice injected subcutaneously at subaxile position in to KM mice—100 mg/kg or 200 mg/kg i.p. for 10 days.	Dose-dependent tumour suppression; enhance body weight; upregulate the level of IL-2, IL-6, and TNF-*α*—increase the serum cytokine levels in tumor-bearing mice.	Deng et al. [15]
Acid (I) and alkali (II) extractible polysaccharides; I comprised of Glc, Fru and Man, whereas II was of Glc and Fru. Glc was the dominant monosaccharide in both (molar percentage of >60%).	Mice—1–21 g/kg, i.g. for 10 days	DIPs I and II could not enhance the cell-mediated immunity and stimulate T cell formation; both enhance macrophages phagocytosis; DIP-II enhance NK cells killing activity; I increase the weight of thymus organ phagocytosis of monocyte; II could restore delayed-type hypersensitivity reaction to dinitrofluorobenzene (DNFB); II also improve natural killer cells activity and splenocytes proliferation.	Hua et al. [6]
Polysaccharides I and II (as above, Ref. [1]): I composed of →1)-Glc-(6→: →1)-Man-(3, 6→ with the ratio of 5.6:1.0, while II was composed of →1)-Glc-(6→: →1)-Man-(3,6→: →1)-Xyl-(5→: →1)-Gal-(3→: →1)-Gal-(6→: with the ratio of 4.9: 15.5: 7.8: 1.0: 5.7.	d-Galactose induced senescence in mice—2.7, 5.4 or 16.2 mL/kg.	Increase SOD and GPx activities	Hua et al. [22]
5-(hydroxymethyl)-2-furfural from the methanol extract	Mushroom tyrosinase assay—oxidation of L-DOPA	Dose dependent inhibition with EC_50_ value of 0.98 mM; noncompetitive inhibitor	Sharma et al. [29]
Partially *O*-acetylated *α*-D-mannan—T-2-HN	Carrageenan-induced edema and scalded edematous hyperalgesia in rats’ hind paws	Display anti-inflammatory effect in both models	Ukai et al. [50]
T4N and T5N: two water soluble glucans: *β*-(1→3)-D-glucan with side branches of *β*-(1→6)-glucosyl units; and T2HN as a (1→3)-*α*-D-mannopyranosyl residue that contain *O*-acetyl group	Sarcoma S180 tumour bearing mice— 5, 10 or 25 mg/kg i.p. for 10 days	T4N and T5N showed antitumor activity at 5 and 10 mg/kg; T2HN showed activity at 25 mg/kg.	Ukai et al. [51]
1→6)-branched (1→3)-*β*-d-glucan (T-5-N), isolated from a sodium hydroxide extract—triple-helical structure in neutral or slightly alkaline solution	Carrageenan-induced edema and scalded edematous hyperalgesia in rats’ hind paws—25 mg/kg i.p.	Anti-inflammatory effect	Hara et al. [52]

Abbreviations: ALP, alkaline phosphatase; ALT, alanine aminotransferase; AST, aspartate aminotransferase; bcl-2, B-cell lymphoma 2; CAT, catalase; CK, creatine kinase; DSS, Dextra Sulfate Sodium; ERK1, extracellular signal–regulated kinase; FOXO, Forkhead box protein O; Fru, fructose; Gal, galactose; Glc, glucose; GSH, glutathione; GPx, glutathione peroxidase; HDL-C, high-density lipoprotein cholesterol; HO-1, Heme oxygenase 1; IFN, interferon; i.g., intragastric route of administration; IκB, inhibitor of κ-B; IL- interleukin; iNOS, inducible nitric oxide synthase; LDH, lactate dehydrogenase; LDL-C; low density lipoprotein cholesterol; LDH, lactate dehydrogenase; LPO, lipid peroxidation; L-DOPA, **L**-3,4-dihydroxyphenylalanine; Man, mannose; MPO, Myeloperoxidase enzyme; NEFA, non-esterified fatty acid; MDA, malondialdehyde; MPO, myeloperoxidase; NEFA, non-esterified fatty acids; NLRP3, nod-like receptor family pyrin domain containing 3; OGTT, oral glucose tolerance test; p.o., intraperitoneal route of administration; ROS, reactive oxygen species; SOD, superoxide dismutase; STAT3, signal transducer and activator of transcription 3; T-AOC, total antioxidant capacity; TBIL, total bilirubin; TC, total cholesterol; TG, triglyceride; TJP-1, Tight junction protein 1; TNF, tumor necrosis factor, Xyl, xylose.
